# Supplementary material for: Combining high-resolution scanning tunnelling microscopy and first-principles simulations to identify halogen bonding
Source: Nat Commun. 2020 Apr 30;11:2103. doi: 10.1038/s41467-020-15898-2 (PMC7192931; doi:10.1038/s41467-020-15898-2)
Supplement: Supplementary file 1 — Supplementary Information [file 41467_2020_15898_MOESM1_ESM.pdf]

Supplementary Information

Combining high-resolution scanning tunnelling microscopy and first-principles simulations to identify halogen bonding

James Lawrence et al.

Combining high-resolution scanning tunnelling microscopy and first-principles simulations to identify halogen bonding

James Lawrence,[1] Gabriele C. Sosso,[1,2,*] Luka Đorđević,[3] Harry Pinfold,[1] Davide Bonifazi,[3,*] Giovanni Costantini[1,*]

[1] Department of Chemistry University of Warwick, Gibbet Hill Road, Coventry CV4 7AL, UK
[2] Centre for Scientific Computing, University of Warwick, Gibbet Hill Road, Coventry CV4 7AL, UK
[3] School of Chemistry, Cardiff University, Park Place Main Building, Cardiff CF10 3AT, UK

* G.Sosso@warwick.ac.uk
* BonifaziD@cardiff.ac.uk; davide.bonifazi@univie.ac.at
* G.Costantini@warwick.ac.uk
† present address: Institute of Organic Chemistry, University of Vienna, Währinger Strasse 38, 1090 Vienna, Austria

Supplementary Information

Contents

Supplementary Figures

- Molecular assemblies

Supplementary Discussion

- Demonstration of the poor fit of the hydrogen bonded assembly over HR-STM images
- Changing the height of the CO tip: Effect on molecular contrast
- CO Clusters on Au(111)
- Comparison between XB and HB 3,9-Br_2PXX assemblies

Supplementary Methods

- Scanning tunnelling microscopy methods
- Density functional theory methods
- Synthetic methods
- X-ray analysis

Supplementary References

Supplementary Figures

Molecular assemblies.

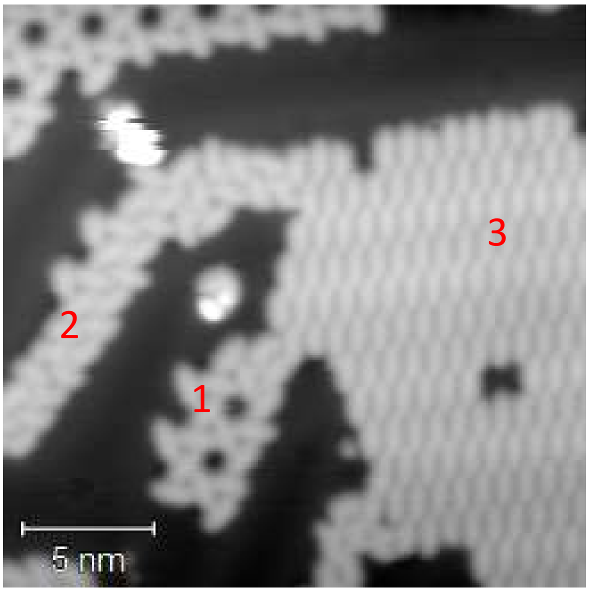

Supplementary Figure 1. STM image of the molecular structures observed by depositing 3,9-Br_2PXX on Au(111) at a coverage close to 1 ML. Three different assemblies are observed to coexist on the surface, corresponding to phases 1, 2 and 3 described in the main paper.

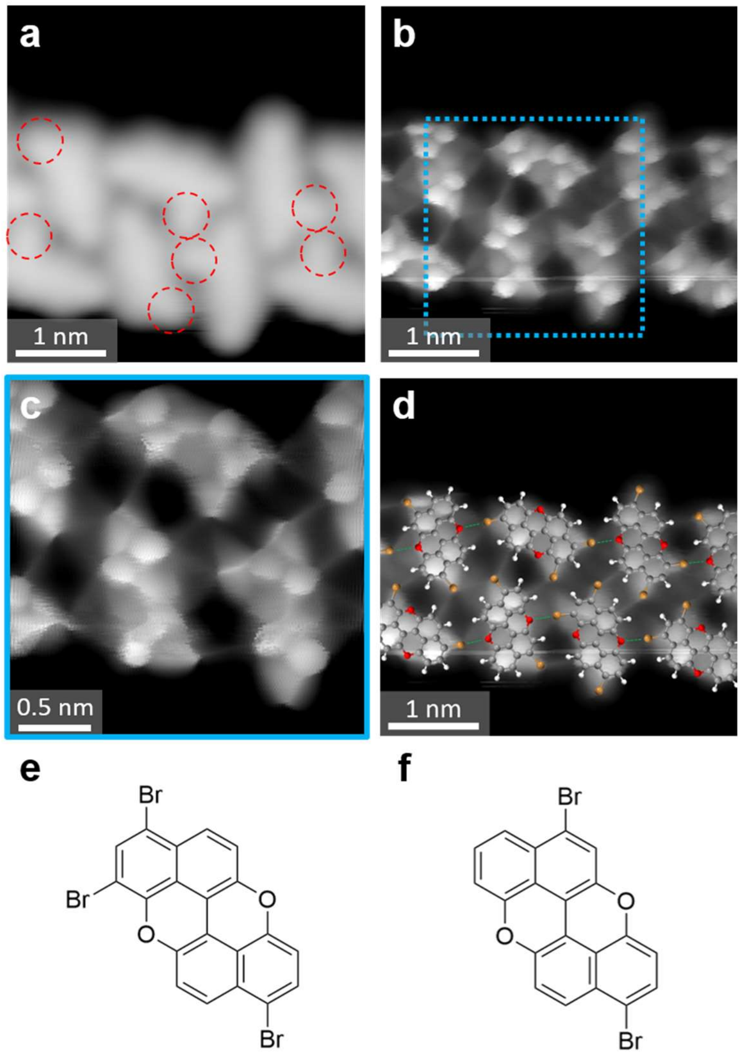

Supplementary Figure 2. HR-STM images of synthetic impurities in the phase 2 assembly. (a) Higher magnification STM image (7 K) of phase 2, showing that some of the molecules have an extra feature at their sides (red dashed circles). (b) – (d) HR-STM images (7 K) of phase 2, with a corresponding model demonstrating that 1,3,9-Br_3-PXX and 2,9-Br_3-PXX molecules participate in the assembly. (e) and (f), structures of 1,3,9-Br_3-PXX and 2,9-Br_3-PXX, respectively.

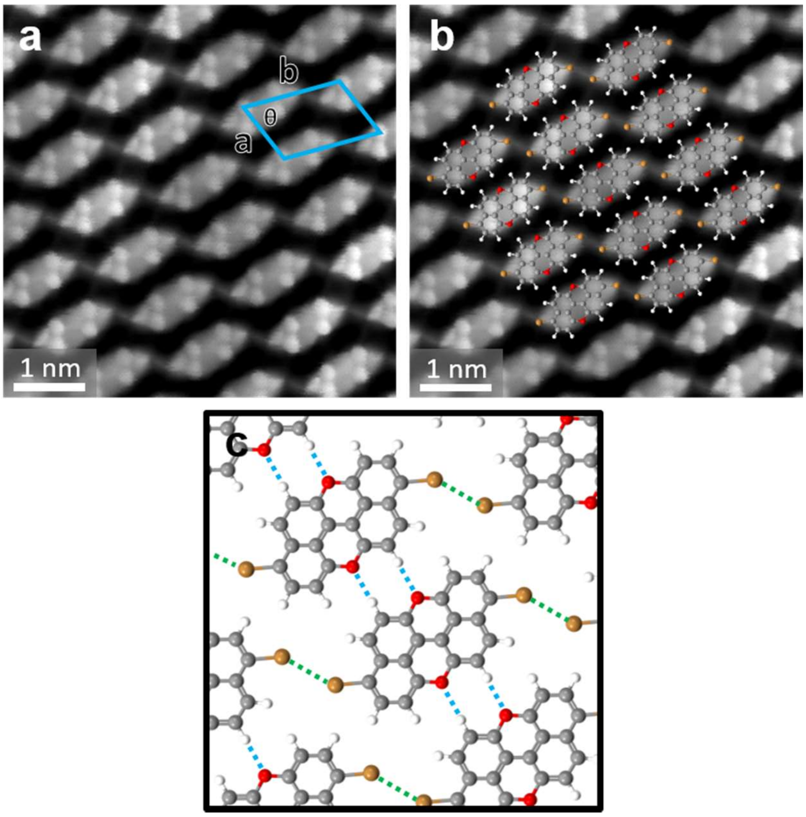

Supplementary Figure 3. HR-STM images of the phase 3 assembly and corresponding model. (a) CO-tip constant height STM image (30 mV) of the high coverage, compact phase 3 assembly, with molecular overlay in (b). Unit cell **a** = 0.83 ± 0.02 nm, **b** = 1.31 ± 0.06 nm, θ = 75 ± 3°. (c) Model of the packing. Molecules interact via hydrogen bonding (blue dashed line, O···H distance of 2.2 ± 0.1 Å) and type I halogen bonding (green dashed line, Br---Br distance 3.6 ± 0.2 Å). Notably, this packing arrangement is essentially identical to that observed in the solid-state and determined by X-ray diffraction analysis (Supplementary Fig. 4).

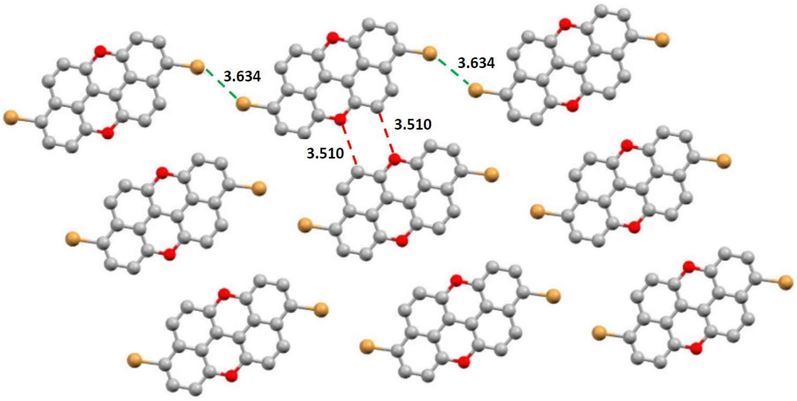

Supplementary Figure 4. Molecular arrangement of 3,9-Br_2PXX in the solid state as obtained from single-crystal X-ray analysis. Space group: P -1. Unit cell: a = 3.8863(3) Å, b = 7.1449(6) Å, c = 13.1979(10) Å. The molecules are organised in a lamellar-like arrangement through a combination of in-plane XB and HB contacts and (interplanar) π- π stacking interactions. Heteroatoms distance of the HB is O···C: 3.510(3) Å, whereas for the XB is Br···Br: 3.634(3) Å. The arrangement is the same as that observed on the surface at high coverage. Notably, the distances of the non-covalent contacts are very similar to those estimated from the STM data.

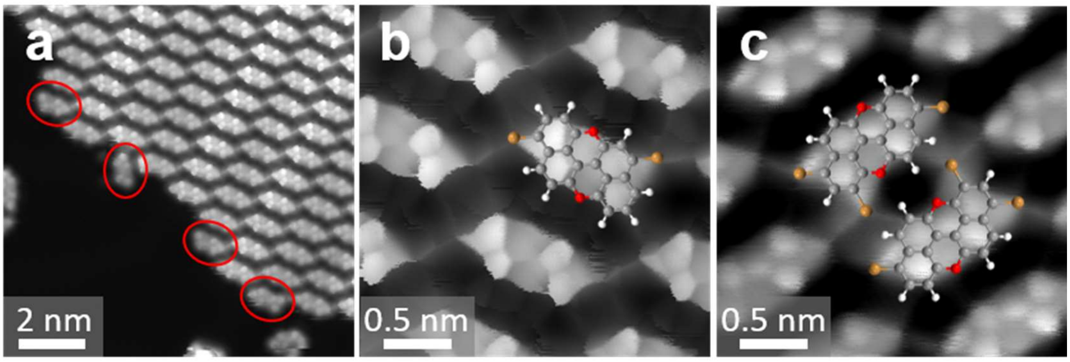

Supplementary Figure 5. HR-STM images of synthetic impurities in the phase 3 assembly. Constant height CO tip images (all 30 mV, 7 K) of defect molecules found in and around the compact island in the high coverage phase 3. (a) Singly de-brominated molecules at the edge of an island. (b) A 2,9-Br_3-PXX isomer defect molecule found within an island. (c) Two adjacent 1,3,9-Br_3-PXX tri-brominated molecules found within an island.

Supplementary Discussion

Demonstration of the poor fit of the hydrogen bonded assembly over HR-STM images. In Supplementary Fig. 6, the alternatively proposed hydrogen bonding structure is overlaid onto the HR-STM image from Fig. 3(a) and compared with the halogen bonded structure, demonstrating the poor fit of the former and the excellent fit of the latter with the experimentally observed molecular features.

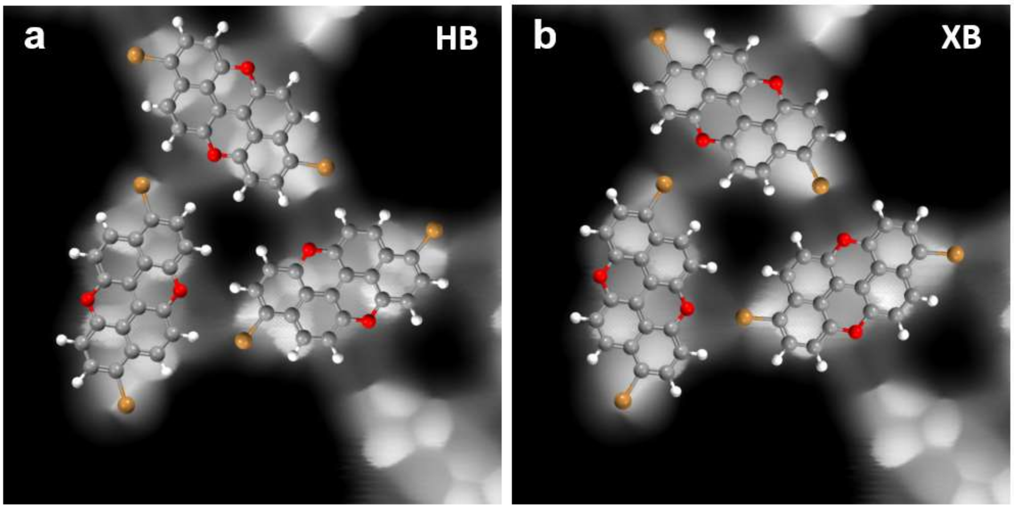

Supplementary Figure 6. Overlaid model of hydrogen bonded assembly onto the experimental HR-STM data from Fig. 3, showing the poor fit with the observed structures when compared to the XB model. (a) HB model and (b) XB model.

Changing the height of the CO tip: Effect on molecular contrast. When performing HR-STM experiments, minor changes in the relative height of the tip can result in distortion of the appearance of the molecules due to the increased repulsion and deflection of the CO tip. Rings become more distorted, and sharp features between the molecules become more apparent.

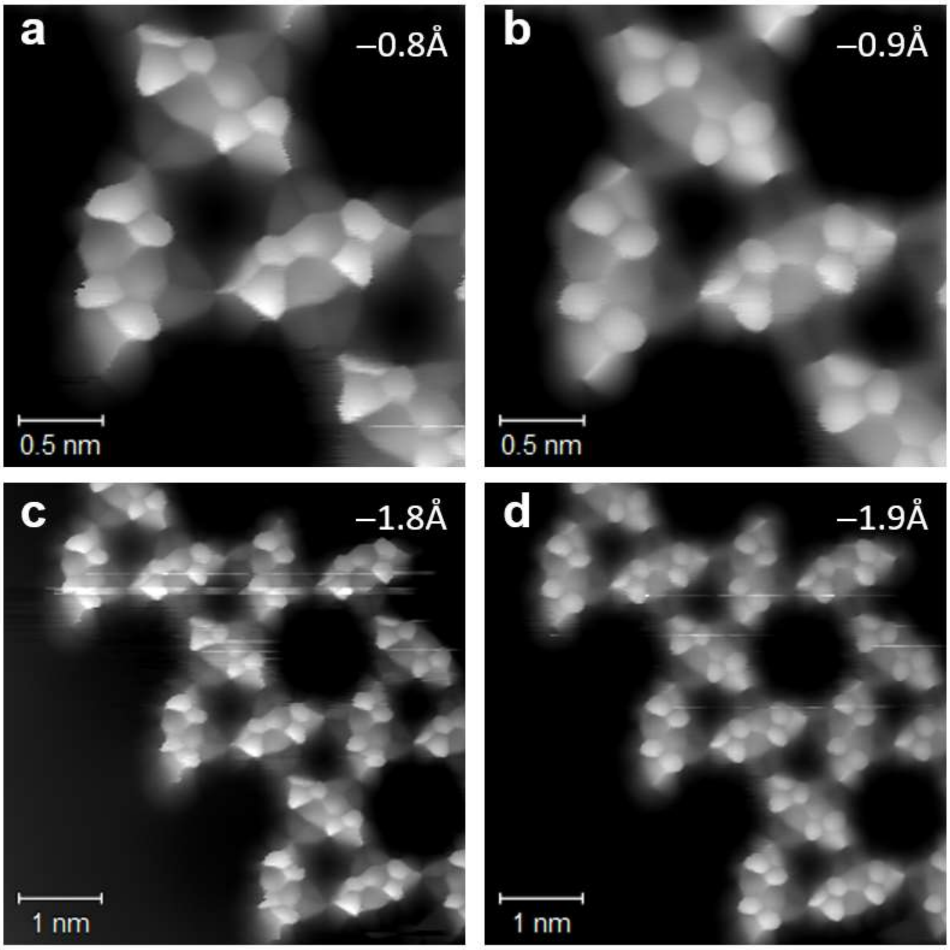

Supplementary Figure 7. HR-STM images of the 3,9-Br_2PXX kagome packing at different tip heights. (a) and (b), same area at decreasing tip-surface distance. (c) and (d), same area at decreasing tip-surface distance. More distortion of the structures is observed with the tip in closer proximity (a less negative tip retract value).

The numbers in the upper right corner of the images in Supplementary Fig. 7 are the relative heights at which these images were acquired. Less negative values are closer to the surface; the absolute height of the tip is unknown. The stabilising current and voltage was the same for both sets of images (1.3 nA, 30 mV). In the case of the larger scale images (bottom row), the tip was stabilised above a bare portion of the surface, and thus had to be moved further away to achieve the same contrast. For the upper row of images, the tip was stabilised over the periphery of a molecule, and thus was retracted less to achieve the same contrast.

CO Clusters on Au(111). CO was typically picked up from the surface by scanning over clusters of CO found after short exposures of the cold (10K) surface to CO (1 x 10^{-7} mbar, 30-60 s). An example of an STM image of a higher coverage of CO clusters co-adsorbed with 3,9-Br_2PXX islands is shown in Supplementary Fig. 8, with one cluster highlighted with a red oval. The CO clusters were found to still be significantly mobile even when scanning at 7K.

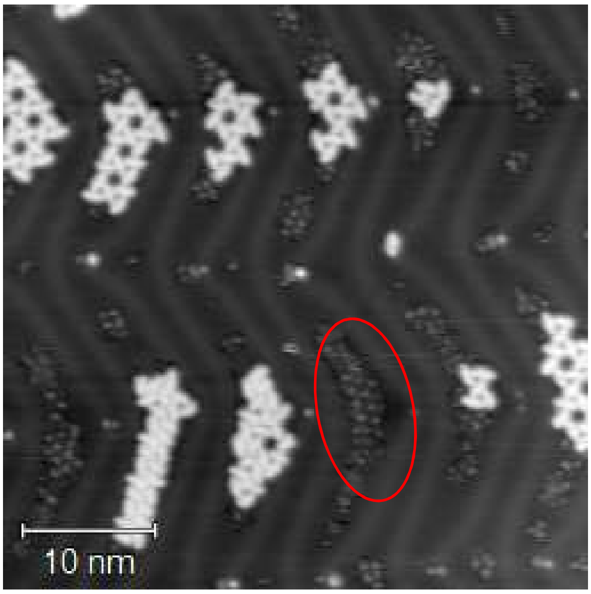

Supplementary Figure 8. STM image of a high coverage of CO clusters alongside 3,9-Br_2PXX islands, with one cluster highlighted with a red oval.

Comparison between XB and HB 3,9-Br_2PXX assemblies. Atomic positions and cell parameters were optimised starting from the experimentally determined values. The resulting maximum force acting on the atoms and the internal pressure of the system were brought down to 0.01 eV/Å and 0.1 bar, respectively. The resulting cell parameters for both the HB and XB assemblies are consistent with the experimental data (see Supplementary Table 1.)

	ΔE_{min} [meV/unit cell]	**a** [Å]	**b** [Å]	θ [deg]	O···H/O···Br [Å]
XB	0.00	22.67 (22.30) ± 0.02	22.60 (22.28) ± 0.02	60.2 (60.0) ± 0.3	3.23 (3.02) ± 0.04
HB	79.0 (97.4) ± 0.2	22.22 (22.01) ± 0.02	22.15 (22.03) ± 0.02	60.3 (60.2) ± 0.3	3.20 (3.06) ± 0.07
Exp.	–	22 ± 1	22 ± 1	60 ± 2	3.1 ± 0.1

Supplementary Table 1. Comparison between XB and HB 3,9-Br_2PXX assemblies, as obtained from DFT calculations using the vdW-DF exchange-correlation functional. Numbers in brackets refer to the results obtained by using the optB88-vdW functional. We report the energy difference between the assemblies, the cell parameters **a**, **b** and θ, and the average O···H/Br distance. Available experimental data (Exp.) from STM measurements (see main text) are also included.

It should be noted that while the usage of different XC functionals has an impact on both the structure and the energetics of the assemblies, the relative trends are robust, and clearly predict that 3,9-Br_2PXX assemblies are held together preferentially by XB interactions as opposed to HB.

As the energy difference reported in Supplementary Table 1 has been evaluated at zero K, it cannot be directly used to determine the Boltzmann factor and thus the expected relative populations of the XB and HB assemblies at room temperature (the temperature at which these molecules are deposited on Au(111)). However, as the variation of ΔE_{min} with temperature is not expected to be huge, using the values in Supplementary Table 1 should still give a reasonable approximation. Doing so, predicts

that 95-98% of the 3,9-Br_2PXX assemblies should be formed by XB interactions, which is in excellent agreement with the 100% observed in the (large but finite) experimental statistics. Finally it should also be considered that the DFT calculations have been performed for free-standing molecules, without considering their interaction with the Au(111) substrate. While this is most probably an excellent approximation (see main paper), we cannot rule out the possibility that the molecule-substrate interactions might have an impact on ΔE_{min}.

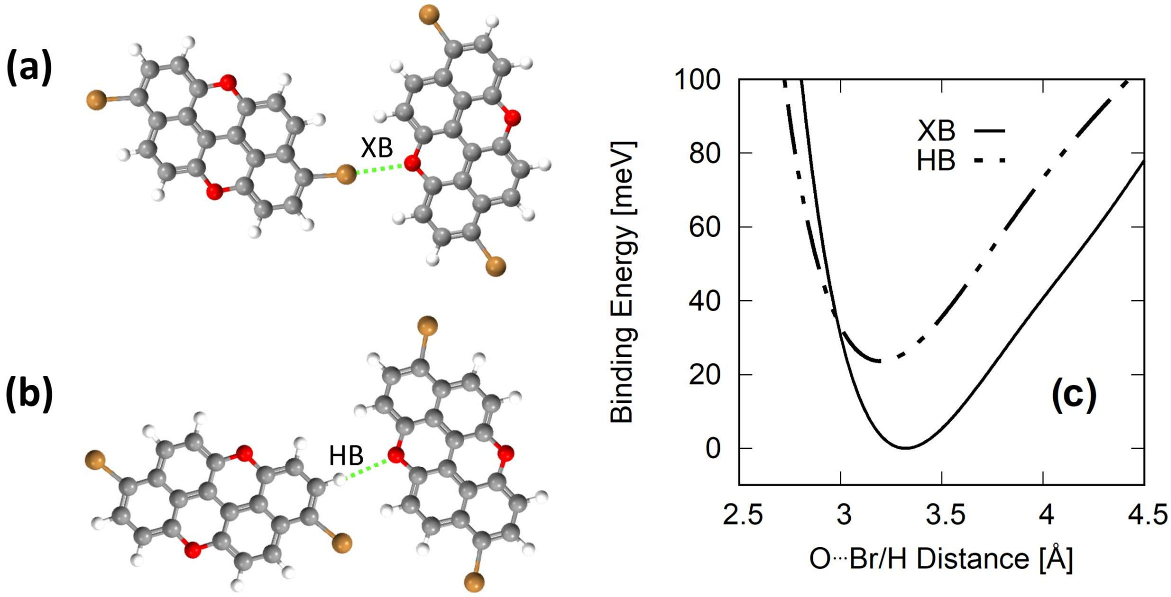

Supplementary Figure 9. DFT calculations of 3,9-Br_2PXX dimers. (a) and (b) Structures of the 3,9-Br_2PXX dimers used to evaluate the binding energies of O···Br (XB) and O···H (HB) interactions, respectively. (c) Binding energy of the XB and HB dimers as a function of the O···Br/H distance.

In order to probe whether the energy difference (see Supplementary Table 1) between the observed XB and an hypothetical HB 3,9-Br_2PXX assembly is due to the particular intermolecular distance of the structure, we have also computed (see Fig. SI.1) the “binding” energy (i.e. the energy corresponding to the XB/HB interaction) of a 3,9-Br_2PXX dimer, held together by either XB or HB interactions – along the direction characterising the experimentally measured 3,9-Br_2PXX assembly. Due to the larger van der Waals radius of Br compared to H, the equilibrium distance for the O···Br interaction is longer than the O···H one (3.32 vs 3.20 Å, respectively); however, the XB interaction is ~ 25 meV stronger than HB at the equilibrium distance, which happens to be very close to the intermolecular distance characterising the observed 3,9-Br_2PXX assembly. Importantly, we find that the XB interaction is stronger than the HB interaction for any given intermolecular distance, thus further strengthening the case for the XB assembly to form. In addition, it is clear that the energy difference between the observed XB and an hypothetical HB 3,9-Br_2PXX assembly (~ 80 meV/unit cell) can be almost entirely ascribed to the three XB interactions within the unit cell (each one of those being ~25 meV stronger than the HB interaction).

Supplementary Methods

Scanning tunnelling microscopy methods. STM experiments were performed on a low temperature STM under ultrahigh vacuum (UHV) conditions. The system has base pressures of 5×10^{-11} mbar in the analysis chamber and 1×10^{-10} mbar in the preparation chamber. The Au(111) single crystal was cleaned with multiple cycles of Ar^+ sputtering (1 keV, 3.5 µA cm^{-2}, 20 mins) and annealing (750 K, 10 mins). 3,9-Br_2PXX was deposited via sublimation (483 K) onto the Au(111) crystal, held at room temperature. The crystal was then cooled to 77 K or 7 K for STM analysis. Standard STM measurements were carried out with bias voltages (applied to the sample) in the range of ±2.0 V and tunnelling currents of 50-200 pA. In order to perform high-resolution STM experiments, CO was leaked into the UHV system (typically for 30-60 s at a pressure of 1×10^{-7} mbar) and consequently adsorbed onto the Au(111) crystal that was held at approximately 10 K in the analysis chamber. To functionalise the STM tip, CO was picked up by either scanning over CO clusters with typical scanning parameters of 100 pA and 1.0-1.5 V or by approaching the tip over the CO clusters by 3-4 Å, starting from initial stabilising conditions of approximately 100 pA and 1.0 V. High-resolution STM images were taken in constant height mode (current channel); after initial stabilisation at 1.3 nA and 30 mV, the height of the tip was adjusted to achieve optimal intramolecular contrast. Moving the tip too close to the molecules resulted in more distortion (Fig. SI.2), whilst scanning with the tip too far away led to a loss in resolution. STM image analysis was performed with WSxM,[3] Gwyddion[4] and LMAPper.[5]

Density functional theory methods. DFT calculations were performed using the mixed Gaussian and Plane-Waves (GPW) method implemented in the CP2K package.[6] As the description of both halogen bonding (XB) and hydrogen bonding (HB) interactions is known to be quite sensitive to the choice of the exchange-correlation (XC) functional,[7] we have used two different fully self-consistent non-local XC functionals, namely vdW-DF[8] and optB88-vdW,[9] to assess the reliability of our results. Goedecker-type pseudopotentials[10] with four, one, six and seven valence electrons for C, H, O and Br respectively have been employed. The Kohn-Sham orbitals were expanded in a Double- Zeta Valence plus Polarization (DZVP) Gaussian-type basis set, while the plane wave cutoff for the finest level of the multi-grid[6] has been set to 400 Ry to efficiently solve the Poisson equation within periodic boundary conditions using the Quickstep scheme.[6] Brillouin zone integration was restricted to the supercell Γ-point. We have found that considering a single unit cell (in-plane dimensions of ~20 Å and containing three 3,9-Br_2PXX molecules, thus totalling 96 atoms), together with a vacuum region of ~20 Å inserted between the periodic replica of the 2D self-assemblies (along the direction normal to the assemblies planes) is sufficient to ensure an accuracy of the resulting total energy of 3 meV/atom.

The simulated HR-STM images have been obtained thanks to the PP-AFM/STM framework of Hapala *et al.*[11] and Krejčí *et al.*[12]. The CO-functionalized tip employed experimentally is approximated by a probe particle bonded to the STM tip: this bond is 4 Å long and characterised by a lateral stiffness of 0.5 N/m. The charge of the probe particle is set equal to zero. As the experimental HR-STM images were obtained in constant height using very low bias voltages, all HR-STM simulated images were calculated as constant height dI/dV maps at the energy of the 3,9-Br_2PXX highest occupied molecular orbital (HOMO). In order to describe the tunnelling process, we have considered the *s* and *p* orbitals of the sample and the p_x and p_y orbitals of the functionalized tip. The Lennard-Jones and electrostatic fields characterizing the sample have been obtained using simple point-charge electrostatics[11] and the equilibrium configuration of the system was used, as obtained via the DFT calculations described above. The electronic density of states and the molecular orbitals of the sample have also been obtained via the CP2K code.

Synthetic method. All chemicals and solvents were purchased from Sigma Aldrich, TCI chemicals, Acros Organics and Fluorochem and were used as received. CuO was dried prior to the reaction at 100 °C. ^{1}H- and ^{13}C-NMR spectra were recorded on *Varian Inova* or *400* MHz NMR spectrometer. Chemical shifts (δ) are reported in parts per million and solvent residual peaks are used as internal standard. High resolution matrix assisted laser desorption ionisation was performed using a Waters Synapt G2-Si time of flight mass spectrometer.

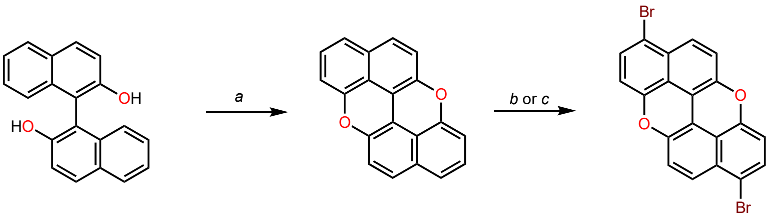

Supplementary Figure 10. Synthesis of PXX and 3,9-Br_2PXX derivative. Reagents and conditions: (a) CuO, 220 °C, 3 h; (b) Br_2, anhydrous CH_2Cl_2, N_2, −78 °C 1h→ rt 2h; (c) *N*-bromomosuccinimide, *o*-dichlorobenzene, 180 °C, 6 h.

***Peri*-xanthenoxanthene** (**PXX**). This compound was prepared according to a modified literature procedure.[13] A mixture of 1,1'-bi-2-naphthol (2.0 g, 7.0 mmol) and CuO (1.2 g, 15.4 mmol) in nitrobenzene (2.0 mL) was stirred at 220 °C for 3 hours, open to air. Then, the nitrobenzene was distilled off and the residue was filtered through a short silica pad with $CHCl_3$ as eluent. Pure product was obtained as bright yellow needles (1.2 g, 61% yield) after sublimation.
^{1}H-NMR (500 MHz, Benzene-*d*$_6$): δ 6.88 (d, *J* = 9.0 Hz, 2H), 6.81 (d, *J* = 2.1 Hz, 2H), 6.80 (s, 2H), 6.68 (d, *J* = 9.0 Hz, 2H), 6.56 (dd, *J* = 5.2, 3.3 Hz, 2H). **^{13}C-NMR** (126 MHz, Benzene-*d*$_6$): δ 153.18, 144.70, 131.76, 127.35, 126.55, 122.12, 120.36, 117.49, 112.00, 108.95. **HRMS** (TOF EI+): found $C_{20}H_{10}O_2$ requires 282.0681, found 282.0682.

3,9-Dibromo-*peri*-xanthenoxanthene (**3,9-Br_2PXX**). This compound was prepared according to two modified literature procedures.[14,15]
Procedure 1. To a stirred suspension of PXX (0.5 g, 1.8 mmol) in anhydrous CH_2Cl_2 (50 mL), at −78 °C under an N_2, was added a solution of bromine (0.6 g, 3.5 mmol) in anhydrous CH_2Cl_2 (10 mL), over 30 minutes. The solution was stirred for 1 hour and left to warm-up to RT, and stirred for 2 more hours. The reaction mixture was poured into a saturated aqueous solution of $NaHSO_3$, and stirred for 1 hour. The mixture was filtered and washed with H_2O, CH_3CH_2OH, and CH_2Cl_2. Product was obtained as yellow needles after re-crystallization from hot *o*-DCB (0.6, 77% yield).
Procedure 2. A stirred suspension of PXX (0.5 g, 1.8 mmol) and NBS (0.6 g, 3.6 mmol) in *o*-DCB was stirred at 180 °C, for 6 hours. The reaction mixture was left to cool down to RT and was diluted with CH_3CH_2OH. The mixture was filtered, washed with CH_3CH_2OH and dried. Product was obtained as yellow needles after re-crystallization from hot *o*-DCB (0.6, 82% yield).
^{1}H-NMR (400 MHz, Benzene-*d*$_6$): δ 7.44 (d, *J* = 9.2 Hz, 2H), 7.04 (d, *J* = 8.2 Hz, 2H), 6.64 (d, *J* = 9.2 Hz, 2H), 6.20 (d, *J* = 8.2 Hz, 2H). **^{13}C-NMR** was not recorded due to the low solubility of this compound. **HRMS** (TOF EI+): found $C_{20}H_8Br_2O_2$ requires 439.8871, found 439.8768. *Note*: by-products, which include the dibromo regioisomer or the tribrominated molecule were observed in STM experiments even after extensive purification procedures – including Soxhlet extraction with THF or benzene and even after 10 re-crystallization cycles using slow cooling from boiling *o*-DCB.

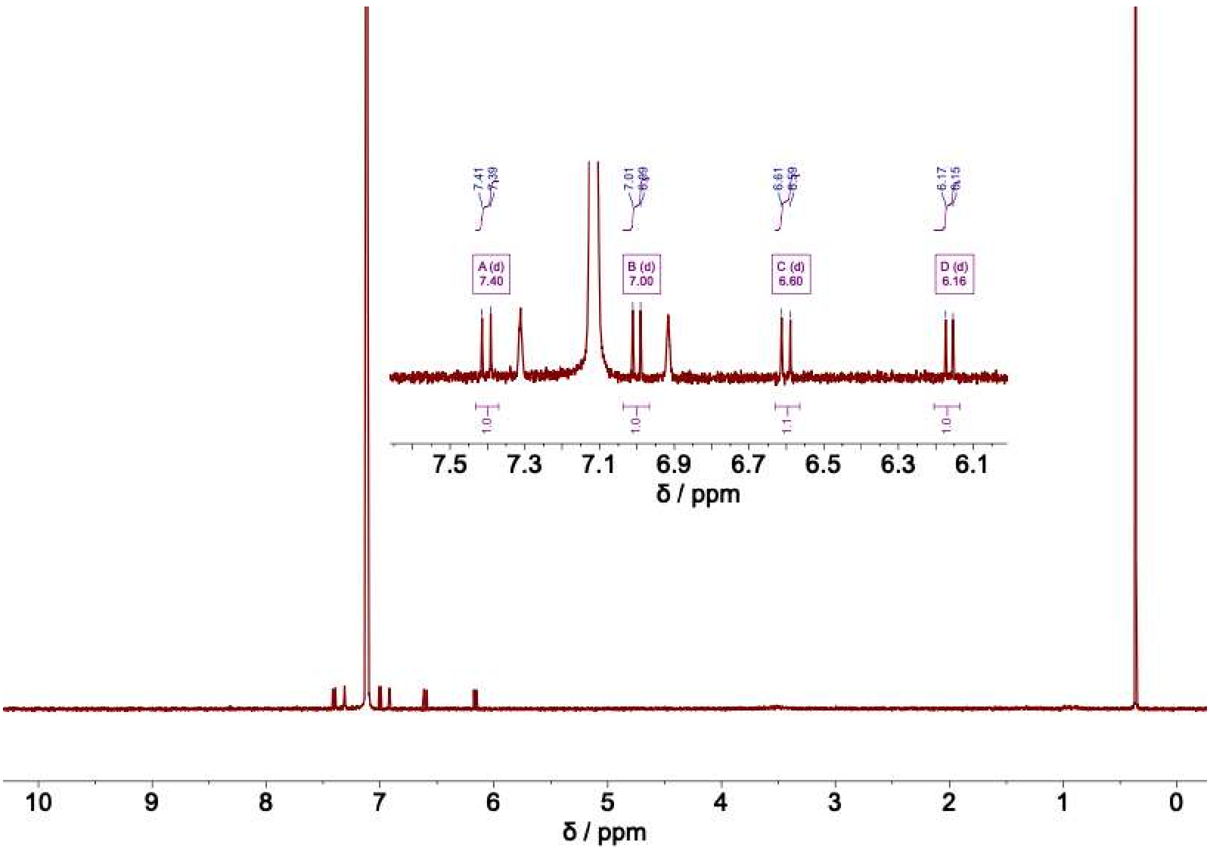

Supplementary Figure 11. ^{1}H-NMR (Benzene-d_6, 298 K) of 3,9-Br_2PXX.

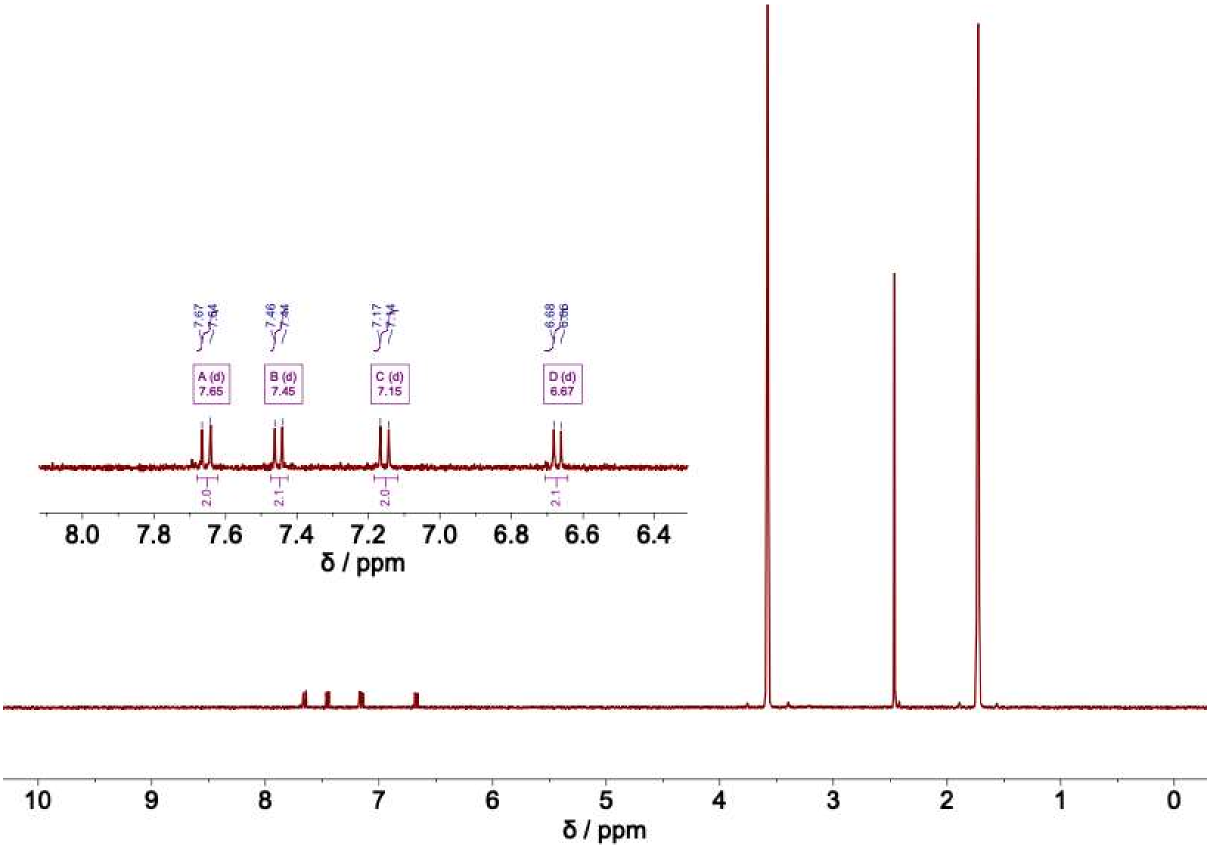

Supplementary Figure 12. ^{1}H-NMR (Tetratdrofuran-d_6, 298 K) of 3,9-Br_2PXX.

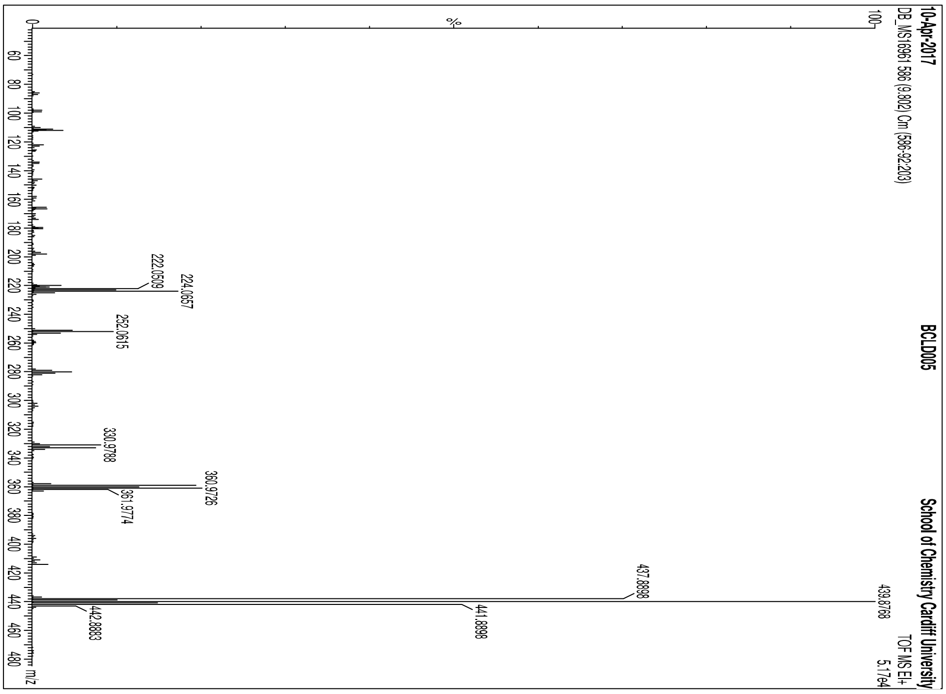

Supplementary Figure 13. Mass spectra of 3,9-Br_2PXX.

Single Mass Analysis

Tolerance = 10.0 PPM / DBE: min = -1.5, max = 50.0
Element prediction: Off

Monoisotopic Mass, Odd and Even Electron Ions
7 formula(e) evaluated with 1 results within limits (all results (up to 1000) for each mass)
Elements Used:
C: 0-20 H: 0-8 O: 0-2 Br: 0-2

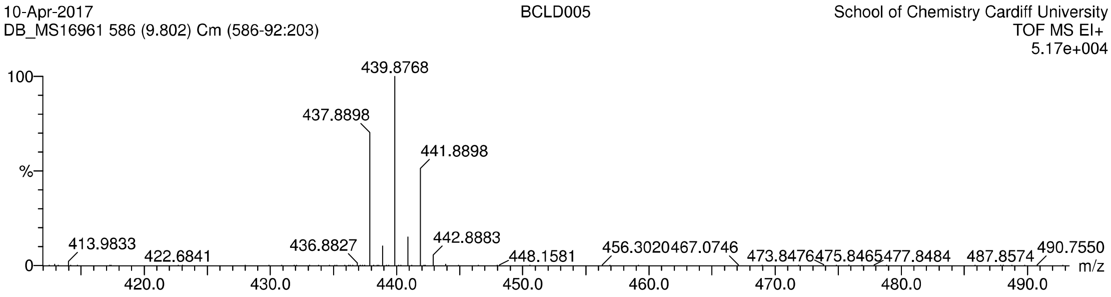

Minimum:				-1.5		
Maximum:		5.0	10.0	50.0		
Mass	Calc. Mass	mDa	PPM	DBE	i-FIT	Formula
437.8898	437.8891	0.7	1.6	16.0	1245.1	C20 H8 O2 Br2

Supplementary Figure 14. Elemental composition report of 3,9-Br_2PXX.

X-ray analysis. Single crystals of 3,9-Br_2PXX were grown by slow evaporation of THF. A single crystal was mounted in Fomblin and diffraction data recorded at Diamond Light Source Beamline I19-1 using Synchrotron radiation (λ = 0.6889 Å) and a photon counting pixel array detector. Measurements were made at 100(2) K with the temperature maintained using an Oxford Cryostream apparatus. Data were collected, integrated and corrected for absorption by an empirical model using DIALS 1.dev.3189-g0030a2438[16] and XIA2 0.5.848-g17d2c5cf.[17] The structure was solved by direct methods (SHELXS)[18] and refined against F^2 within SHELXL.[19] A summary of crystallographic data are available as further supporting information and the structure has been deposited with the Cambridge Structural Database (CCDC deposition number 1901257). These data can be obtained free of charge from The Cambridge Crystallographic Data Centre via www.ccdc.cam.ac.uk/data_request/cif.

Supplementary Table 2. Crystal data and structure refinement for 3,9-Br_2PXX.

Identification code	shelx	
Empirical formula	C20 H8 Br2 O2	
Formula weight	440.08	
Temperature	100(2) K	
Wavelength	0.6889 ≈	
Crystal system	Triclinic	
Space group	P -1	
Unit cell dimensions	a = 3.8863(3)≈	a= 95.852(7)∞.
	b = 7.1449(6)≈	b= 95.766(6)∞.
	c = 13.1979(10) ≈	g = 96.410(6)∞.
Volume	359.95(5) ≈3	
Z	1	
Density (calculated)	2.030 Mg/m3	
Absorption coefficient	5.213 mm-1	
F(000)	214	
Crystal size	0.111 x 0.026 x 0.010 mm3	
Theta range for data collection	1.513 to 35.798∞.	
Index ranges	-6<=h<=6, -11<=k<=11, -21<=l<=21	
Reflections collected	7022	
Independent reflections	3209 [R(int) = 0.0513]	
Completeness to theta = 24.415∞	98.5 %	
Absorption correction	Empirical	
Max. and min. transmission	1.0 and 0.998883499336	
Refinement method	Full-matrix least-squares on F2	
Data / restraints / parameters	3209 / 0 / 109	
Goodness-of-fit on F2	1.003	
Final R indices [I>2sigma(I)]	R1 = 0.0456, wR2 = 0.1274	
R indices (all data)	R1 = 0.0539, wR2 = 0.1317	
Extinction coefficient	n/a	
Largest diff. peak and hole	3.142 and -1.308 e.≈-3	

Supplementary Table 3. Atomic coordinates (x 104) and equivalent isotropic displacement parameters (≈^{2}x 103) for 3,9-Br_2PXX. U(eq) is defined as one third of the trace of the orthogonalized Uij tensor.

	x	y	z	U(eq)
C(1)	7376(6)	3594(4)	6557(2)	19(1)
C(2)	8421(7)	3159(4)	7524(2)	24(1)
C(3)	8040(7)	4459(4)	8390(2)	25(1)
C(4)	6638(6)	6104(4)	8261(2)	22(1)
C(5)	5456(6)	6582(4)	7276(2)	20(1)
C(6)	3909(6)	8244(4)	7073(2)	21(1)
C(7)	2818(6)	8593(4)	6089(2)	21(1)
C(8)	3267(6)	7285(4)	5255(2)	19(1)
C(9)	4762(6)	5669(3)	5420(2)	17(1)
C(10)	5863(6)	5289(4)	6420(2)	18(1)
O(1)	7870(5)	2341(3)	5727(1)	21(1)
Br(1)	6295(1)	7793(1)	9440(1)	28(1)

Supplementary Table 4. Anisotropic displacement parameters (≈^{2}x 10^3)for 3,9-Br_2PXX. The anisotropic displacement factor exponent takes the form: -2p2[h^2a^{*2}U11 + ... + 2 h k a* b* U^{12}]

	U11	U22	U33	U23	U13	U12
C(1)	15(1)	28(1)	15(1)	6(1)	2(1)	2(1)
C(2)	22(1)	33(1)	18(1)	10(1)	0(1)	4(1)
C(3)	21(1)	38(1)	19(1)	9(1)	2(1)	4(1)
C(4)	16(1)	35(1)	15(1)	5(1)	2(1)	2(1)
C(5)	15(1)	30(1)	15(1)	3(1)	4(1)	2(1)
C(6)	18(1)	30(1)	17(1)	2(1)	3(1)	4(1)
C(7)	17(1)	27(1)	21(1)	4(1)	3(1)	4(1)
C(8)	15(1)	26(1)	17(1)	7(1)	2(1)	3(1)
C(9)	15(1)	24(1)	15(1)	5(1)	3(1)	3(1)
C(10)	13(1)	27(1)	13(1)	4(1)	2(1)	2(1)
O(1)	24(1)	26(1)	16(1)	6(1)	2(1)	7(1)
Br(1)	23(1)	48(1)	14(1)	-1(1)	1(1)	10(1)

Supplementary Table 5. Bond lengths [≈] and angles [∞] for 3,9-Br_2PXX.

C(1)-C(2)	1.376(3)
C(1)-O(1)	1.385(3)
C(1)-C(10)	1.423(4)
C(2)-C(3)	1.428(4)
C(2)-H(2)	0.9500
C(3)-C(4)	1.368(4)
C(3)-H(3)	0.9500
C(4)-C(5)	1.422(3)
C(4)-Br(1)	1.895(3)
C(5)-C(10)	1.418(4)
C(5)-C(6)	1.427(4)
C(6)-C(7)	1.381(3)
C(6)-H(6)	0.9500
C(7)-C(8)	1.408(4)
C(7)-H(7)	0.9500
C(8)-C(9)	1.376(3)
C(8)-O(1)#1	1.388(3)
C(9)-C(10)	1.410(3)
C(9)-C(9)#1	1.428(5)
C(2)-C(1)-O(1)	117.9(2)
C(2)-C(1)-C(10)	120.7(2)
O(1)-C(1)-C(10)	121.4(2)
C(1)-C(2)-C(3)	118.9(3)
C(1)-C(2)-H(2)	120.5
C(3)-C(2)-H(2)	120.5
C(4)-C(3)-C(2)	120.5(2)
C(4)-C(3)-H(3)	119.7
C(2)-C(3)-H(3)	119.7
C(3)-C(4)-C(5)	122.2(2)
C(3)-C(4)-Br(1)	118.39(18)
C(5)-C(4)-Br(1)	119.4(2)
C(10)-C(5)-C(4)	116.9(2)
C(10)-C(5)-C(6)	117.2(2)
C(4)-C(5)-C(6)	125.9(2)
C(7)-C(6)-C(5)	122.1(2)
C(7)-C(6)-H(6)	119.0
C(5)-C(6)-H(6)	119.0
C(6)-C(7)-C(8)	119.4(2)
C(6)-C(7)-H(7)	120.3
C(8)-C(7)-H(7)	120.3
C(9)-C(8)-O(1)#1	121.4(2)
C(9)-C(8)-C(7)	120.2(2)
O(1)#1-C(8)-C(7)	118.4(2)
C(8)-C(9)-C(10)	121.0(2)
C(8)-C(9)-C(9)#1	120.8(3)
C(10)-C(9)-C(9)#1	118.2(3)
C(9)-C(10)-C(5)	120.1(2)
C(9)-C(10)-C(1)	119.1(2)
C(5)-C(10)-C(1)	120.7(2)
C(1)-O(1)-C(8)#1	119.0(2)

Symmetry transformations used to generate equivalent atoms:
#1 -x+1,-y+1,-z+1

Supplementary References

1. Bondi, A. Van der waals volumes and radii. *J. Phys. Chem.* **68**, 441-451 (1964).
2. Desiraju, G. R. *et al.* Definition of the halogen bond (IUPAC Recommendations 2013). *Pure Appl. Chem* **85**, 1711-1713 (2013).
3. Horcas, I. *et al.* WSXM: a software for scanning probe microscopy and a tool for nanotechnology. *Rev. Sci. Instrum.* **78**, 013705 (2007).
4. Nečas, D. & Klapetek, P. Gwyddion: an open-source software for SPM data analysis. *Open Phys.* **10**, 181-188 (2012).
5. Perdigão, L. M. A. *LMAPper - The SPM and Mol Viewer*, https://sourceforge.net/projects/spm-and-mol-viewer/
6. VandeVondele, J. *et al.* Quickstep: Fast and accurate density functional calculations using a mixed Gaussian and plane waves approach. *Comput. Phys. Commun.* **167**, 103-128 (2005).
7. Kolář, M. H. & Hobza, P. Computer Modeling of Halogen Bonds and Other σ-Hole Interactions. *Chem. Rev.* **116**, 5155-5187 (2016).
8. Dion, M., Rydberg, H., Schroder, E., Langreth, D. C. & Lundqvist, B. I. Van der Waals density functional for general geometries. *Phys. Rev. Lett.* **92**, 246401 (2004).
9. Klimes, J., Bowler, D. R. & Michaelides, A. Chemical accuracy for the van der Waals density functional. *J. Phys. Condens. Matter* **22**, 022201 (2010).
10. Goedecker, S., Teter, M. & Hutter, J. J. P. R. B. Separable dual-space Gaussian pseudopotentials. *Phys. Rev. B* **54**, 1703 (1996).
11. Hapala, P. *et al.* Mechanism of high-resolution STM/AFM imaging with functionalized tips. *Phys. Rev. B* **90**, 085421 (2014).
12. Krejčí, O., Hapala, P., Ondráček, M. & Jelínek, P. Principles and simulations of high-resolution STM imaging with a flexible tip apex. *Phys. Rev. B* **95**, 045407 (2017).
13. Pummerer, R., Prell, E. & Rieche, A. Darstellung von binaphthylendioxyd. *Ber. Dtsch. Chem. Ges.* **59**, 2159-2161 (1926).
14. Kobayashi, N., Sasaki, M. & Nomoto, K. Stable peri-Xanthenoxanthene Thin-Film Transistors with Efficient Carrier Injection. *Chem. Mater.* **21**, 552-556 (2009).
15. Pat. Appl.WO2008/011964A1, 2008.
16. Winter, G. xia2: an expert system for macromolecular crystallography data reduction. *J. Appl. Cryst.* **43**, 186-190 (2010).
17. Winter, G. *et al.* DIALS: implementation and evaluation of a new integration package. *Acta Crystallogr. Sect. D. Biol. Crystallogr.* **74**, 85-97 (2018).
18. Sheldrick, G. M. A short history of SHELX. *Acta Crystallogr. A* **64**, 112-122 (2008).
19. Sheldrick, G. M. Crystal structure refinement with SHELXL. *Acta Crystallogr. Sect. C: Cryst. Struct. Commun.* **71**, 3-8 (2015).
